# A New Work by the Late Prof. Dunglison

**Published:** 1872-02

**Authors:** 


### A New Work by the late Prof. Dunglison.

Messrs. Lindsay & Blakiston announce that they will shortly publish a work entitled History of Medicine, by the late Prof. Robley Dunglison, M.D., L.L.D. collected and arranged from the original manuscript by his son Richard J Dunglison, M. D. This work will be looked forward to with interest and received with pleasure by the Medical Profession of the U. S. The book will no doubt contain much valuable information, and will doubtless be an interesting narrative of events in the History of Medicine, coming as it does from the able pen of Prof. Dunglison.

